# Combined reduction in the expression of MCL-1 and BCL-2 reduces organismal size in mice

**DOI:** 10.1038/s41419-020-2376-5

**Published:** 2020-03-13

**Authors:** Francine Ke, Graeme I. Lancaster, Stephanie Grabow, Andrew J. Murphy, Andreas Strasser

**Affiliations:** 1 The Walter and Eliza Hall Institute of Medical Research, Melbourne, VIC 3052 Australia; 2 The Department of Medical Biology, The University of Melbourne, Melbourne, VIC 3052 Australia; 3 Baker Heart and Diabetes Institute, Melbourne, VIC 3004 Australia; 4 Department of Immunology, Monash University, Melbourne, VIC 3004 Australia; 5 Present Address: Blueprint Medicines, Cambridge, MA 02139 USA

**Keywords:** Hormones, Cell death

## Abstract

The intrinsic apoptotic pathway is controlled by the BCL-2 family of proteins, which exhibit either a pro-death or pro-survival function. Gene knockout studies revealed that different pro-survival BCL-2 proteins are critical for the survival of distinct cell types, although overlapping functions amongst such proteins have also been identified. In the process of studying mice lacking single alleles of *Mcl-1* (*Mcl-1*^+/−^)*, Bcl-2* (*Bcl-2*^+/−^), or both in combination (*Mcl-1*^+/−^*Bcl-2*^+/−^), we observed that *Mcl-1*^+/−^*Bcl-2*^+/−^ mice weighed less when compared with their wild-type littermates as they aged. Body composition analysis demonstrated that while fat mass was similar to wild-type controls, lean mass was significantly reduced in *Mcl-1*^+/−^, *Bcl-2*^+/−^, and, most strikingly in *Mcl-1*^+/−^*Bcl-2*^+/−^ mice. The weights of several tissues including the heart, tibialis anterior, and kidney were likewise reduced in *Mcl-1*^+/−^*Bcl-2*^+/−^ mice. When lean mass and specific tissue weights were expressed relative to body weight, these differences were no longer significant, indicating that that *Mcl-1*^+/−^*Bcl-2*^+/−^ mice, and to a lesser extent *Mcl-1*^+/−^ and *Bcl-2*^+/−^ mice, are smaller than their wild-type counterparts. Consistently, the anal-naso length was reduced in *Mcl-1*^+/−^*Bcl-2*^+/−^ mice. While minor reductions in size were observed in female *Mcl-1*^+/−^*Bcl-2*^+/−^ mice, these effects were most prominent in males. Notably, *Mcl-1*^+/−^*Bcl-2*^+/−^ males had markedly smaller testes even after accounting for differences in body weight. Collectively, these data reveal that combined loss of a single allele of *Mcl-1* and *Bcl-2*, while not overtly impairing organismal development, leads to a reduction in animal size.

## Introduction

The intrinsic (also called the BCL-2-regulated or mitochondrial) apoptotic pathway, is a physiological process that is regulated by the BCL-2 family of proteins^1,2^. It contributes to diverse functions, including maintaining cellular homeostasis, sculpting the embryo by removing excess structures during development, and eliminating damaged or infected cells^1–3^. All members of the BCL-2 family share domains of homology (called BCL-2 homology (BH) domain) and can be divided into two groups based on their function to promote cell survival (BCL-2, BCL-XL, BCL-W, MCL-1, BFL-1/A1), or cell death. The pro-apoptotic members are further classified into two groups—the multi-BH domain proteins (BAX, BAK, BOK) that execute cell death and the BH3-only proteins that contain only the BH3 domain (BAD, BID, BIK, BIM, BMF, HRK, NOXA, PUMA) and act as initiators of apoptosis signalling^1,2,4^.

In response to cytotoxic stimuli, such as DNA damage, or developmental cues, the expression of BH3-only proteins is induced transcriptionally or post-transcriptionally^5^. These initiators of apoptosis bind to and inhibit the pro-survival BCL-2 proteins thereby liberating BAX and BAK to permeabilise the mitochondrial outer membrane (MOMP). This causes the release of apoptogenic proteins (e.g., cytochrome c) from the mitochondria that activate caspases to orchestrate cellular demolition^1,2,4^. Certain BH3-only proteins, such as BIM and PUMA, have been reported to also be able to directly activate BAX/BAK to induce MOMP and apoptosis^1,2,4^.

Earlier work aimed to determine the roles of various BCL-2 family members in cancer and embryonic development through the study of single, as well as multiple gene knockout mice^3,6^. Such studies revealed that systemic loss of either pro-survival MCL-1^7^ or BCL-XL^8^ resulted in embryonic lethality, while the loss of BCL-2 gave rise to runty animals that succumbed to polycystic kidney disease at a young age (4–7 weeks post-birth)^9,10^. We examined the overlapping roles of different pro-survival BCL-2 family members by generating *Mcl-1*^+/−^*Bcl-x*^+/−^, *Mcl-1*^+/−^*Bcl-2*^+/−^ and *Bcl-x*^+/−^*Bcl-2*^+/−^ mice. While the combined loss of single alleles of *Mcl-1* and *Bcl-x* disrupted normal embryogenesis and gave rise to animals with severe craniofacial abnormalities, with most dying soon after or even before birth, these defects were prevented by the deletion of a single allele of the pro-apoptotic BH3-only protein, BIM^6^. In contrast, *Mcl1*^+/−^*Bcl-2*^+/−^ and *Bcl-x*^+/−^*Bcl-2*^+/−^ mice appeared grossly normal and were long-lived^6^.

However, in the course of maintaining these colonies, we observed that double heterozygote *Mcl-1*^+/−^*Bcl-2*^+/−^ mice gained less weight as they aged in comparison to their wild-type (WT) counterparts. This reduced weight gain was due to reduced growth, with male *Mcl-1*^+/−^*Bcl-2*^+/−^ mice displaying lower lean mass, tissue weights, and a commensurate decrease in body length. However, their fat mass remained normal and no overt developmental anomalies were observed in comparison to WT animals.

## Results

### Mice lacking a single allele of *Mcl-1* and a single allele of *Bcl-2* are smaller in size compared to WT animals

**Fig. 1:**
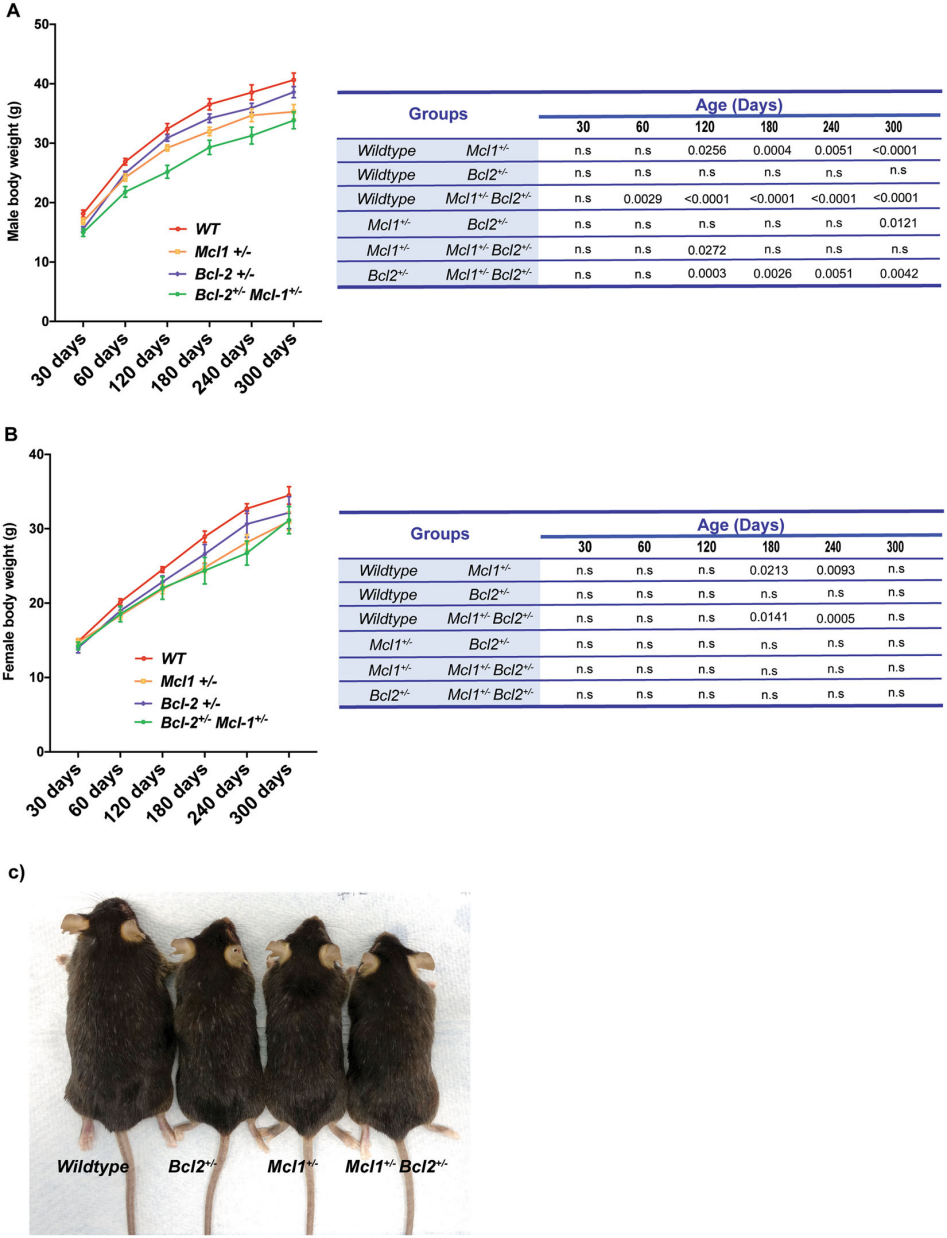
*Mcl-1*^+/^*Bcl-2*^+/−^ mice are smaller in size compared with WT mice. **a**, **b** Body weights of WT, *Mcl-1*^+/−^, *Bcl-2*^+/−^ and *Mcl-1*^+/^*Bcl-2*^+/−^ male (*n* = 7–15 mice per genotype) and female (*n* = 10–13 mice per genotype). Mice were observed for over 300 days. Graphs depict the mean ± S.E.M. of mice weights over time and data were analysed using two-way ANOVA. Statistically significant differences between groups are indicated in the corresponding tables. **c** Representative image of age-matched male mice (280 days) from all 4 genotypes examined.

Over the course of breeding *Mcl-1*^+/−^ and *Bcl-2*^+/−^ animals, we noticed that mice lacking single alleles of *Mcl-1* or *Bcl-2*, and in particular the *Mcl-1*^+/−^*Bcl-2*^+/−^ double heterozygotes, gained less weight and were smaller than WT controls (Fig. 1a–c).

**Fig. 2:**
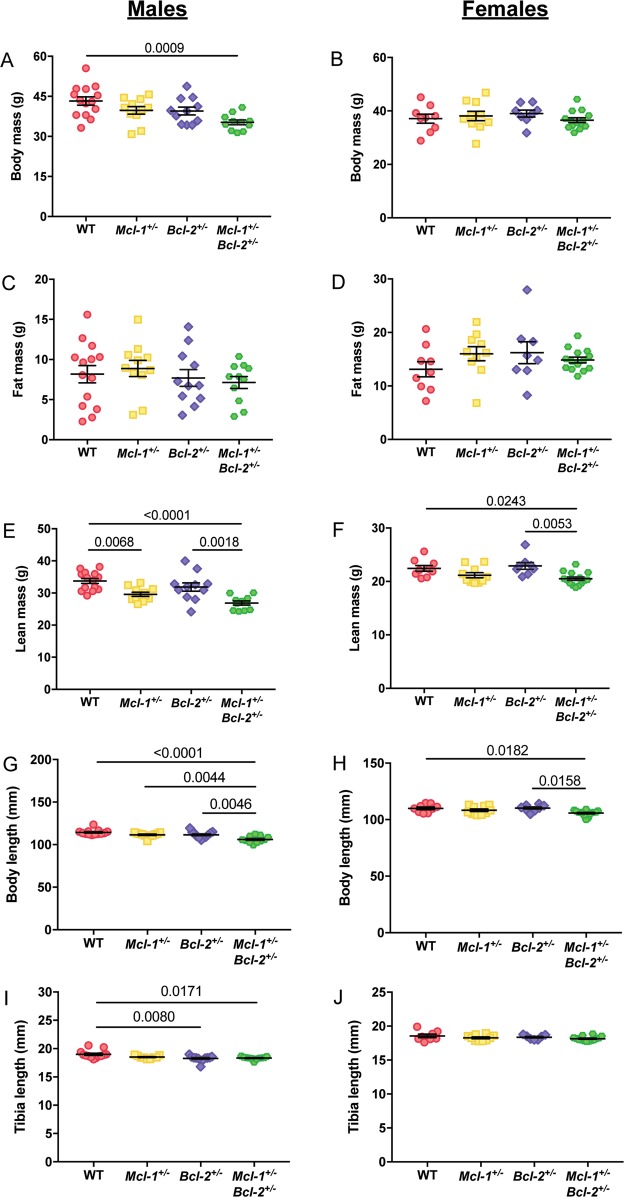
*Mcl-1*^+/^*Bcl-2*^+/−^ mice are shorter in length and have a lower lean mass compared to WT controls. EchoMRI was used to determine the (**a**, **b**) body mass, (**c**, **d**) fat mass, and (**e**, **f**) lean mass of male (*n* = 11–14 animals per genotype, mean age of 509 days) and female (*n* = 8–14 animals per genotype, mean age of 517 days) mice. **g**, **h** Body length and (**i**, **j**) tibia length were also measured. Data represent mean ± S.E.M. and were analysed using one-way ANOVA comparing the mean of each group to every other mean. *P* ≤ 0.05 indicate statistical significance.

We initially considered that *Mcl-1*^+/−^*Bcl-2*^+/−^ mice may be protected from the development of age-related obesity. Therefore, to determine whether the observed weight differences could be attributed to alterations in fat mass, we assessed body composition by EchoMRI^11^ to measure body mass (Fig. 2a, b), fat mass (Fig. 2c, d), and lean mass (Fig. 2e, f) in an independent cohort of aged male and female WT, *Mcl-1*^+/−^, *Bcl-2*^+/−^ and *Mcl-1*^+/−^*Bcl-2*^+/−^ animals. In contrast to our hypothesis, no differences in fat mass were observed between the genotypes examined (Fig. 2a, b). However, a striking difference in lean mass was evident between WT and *Mcl-1*^+/−^*Bcl-2*^+/−^ mice, which was more pronounced in males than females (compare Fig. 2e, f). Consistent with these data, we explored the overall size of the mice and noted a significant decrease in measured body length (naso-anal length) and tibia length in male *Mcl-1*^+/−^*Bcl-2*^+/−^ animals when compared with WT controls (Fig. 2g–j).

**Fig. 3:**
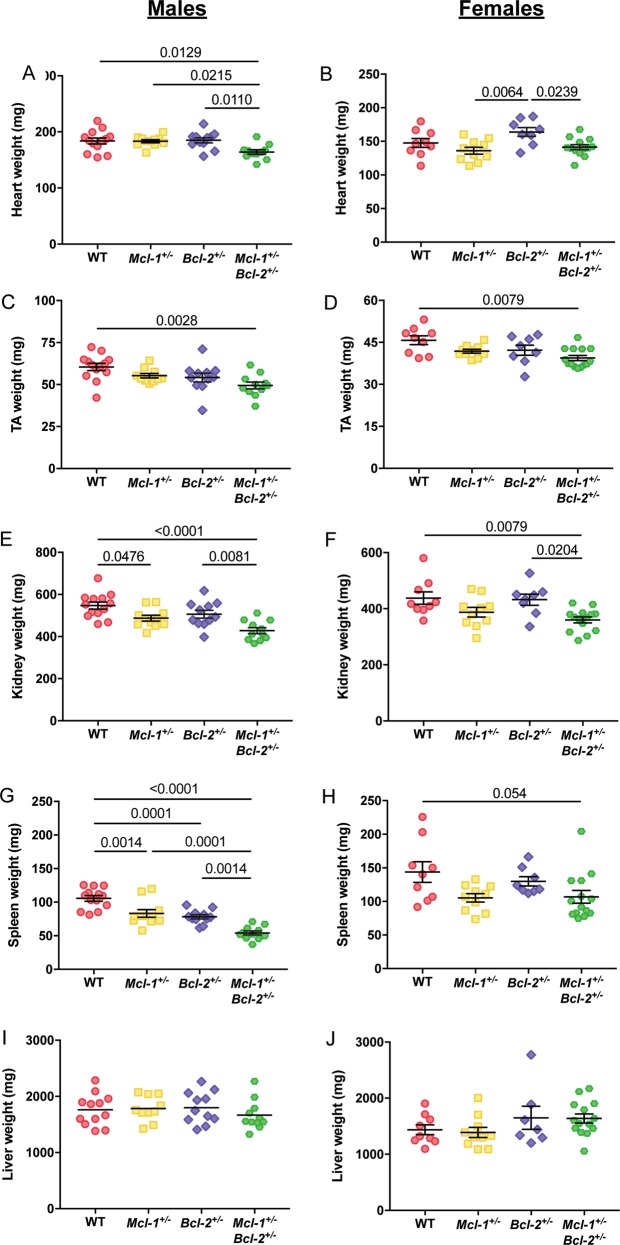
Organ weights of male and female animals. Major organs including the (**a**, **b**) heart, (**c**, **d**) TA muscle, (**e**, **f**) kidneys, (**g**, **h**) spleen and (**i**, **j**) liver were weighed. Data represent mean ± S.E.M. of organ weights from male or female mice and were analysed by one-way ANOVA. *P* ≤ 0.05 indicate statistical significance.

To further assess the reduced lean mass phenotype observed, we explored the weights of several major organs. Heart (Fig. 3a, b), tibialis anterior muscle (TA) (Fig. 3c, d), kidney (Fig. 3e, f) and spleen weights (Fig. 3g, h) were also all significantly reduced in male *Mcl-1*^+/−^*Bcl-2*^+/−^ animals when compared with WT controls. We observed a similar trend in *Mcl-1*^+/−^, as well as *Bcl-2*^+/−^ single heterozygote male mice, although the magnitude of these changes was smaller in comparison to the *Mcl-1*^+/−^*Bcl-2*^+/−^ double heterozygote animals. Female *Mcl-1*^+/−^*Bcl-2*^+/−^ mice also displayed a significant reduction in body length (Fig. 2h), TA (Fig. 3d), kidney (Fig. 3f) and spleen weight (Fig. 3h) compared to WT controls. No significant differences in liver weights were observed across the genotypes in mice of both sexes (Fig. 3i, j). From kidney histology sections, we counted the number of nuclei within proximal and distal tubules per field from images of the renal cortex in multiple WT and *Mcl-1*^+/−^*Bcl-2*^+/−^ male mice. The numbers of nuclei/field were not different between genotypes (data not shown), indicating that the above described reductions in organ weights are likely due to reductions in the total number of cells per organ rather than decreases in cell size.

**Fig. 4:**
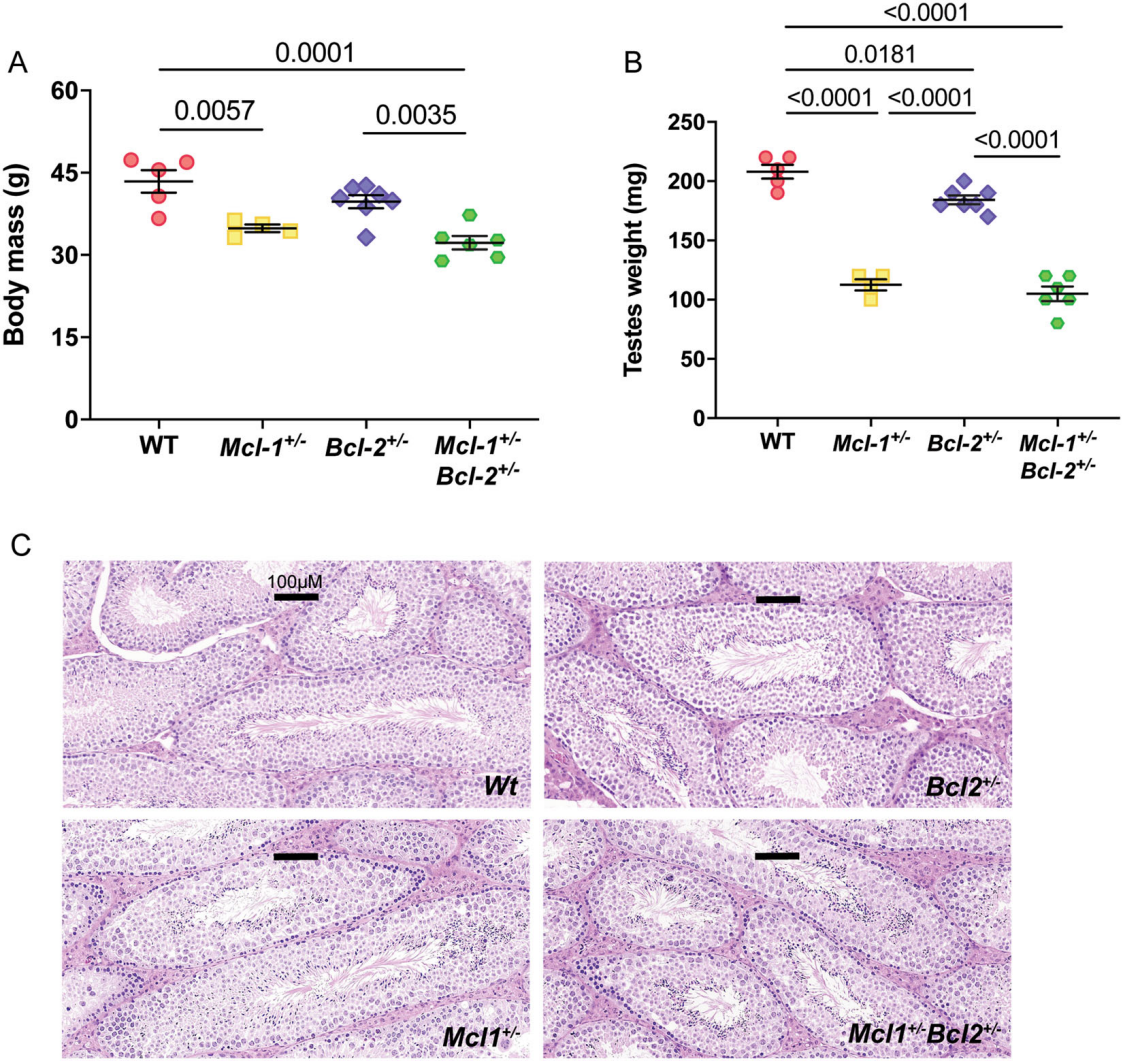
Loss of one allele of *Mcl-1* or *Bcl-2* leads to decreased testicle weights in mice. **a** Body mass and (**b**) testes weights were measured in a cohort of age-matched males (*n* = 4–7 mice per genotype, mean age = 278 days). Data represent mean ± S.E.M. of weight and were analysed by one-way ANOVA. Statistically significant differences (*P* ≤ 0.05) are indicated in the graphs. **c** Representative images of haematoxylin and eosin stained histological sections of the testes from mice of the indicated genotypes. Images are shown at ×14 magnification. Black bars in all histology images represent 100 μm.

In the initial cohort of aged males studied, testes weights were not included in our analyses. However, this information was subsequently recorded along with body mass (Fig. 4a) in a second batch of younger male mice (mean age = 278 days). In this cohort we again observed that *Mcl-1*^+/−^*Bcl-2*^+/−^ males consistently displayed reduced weight in comparison with WT controls. Furthermore, *Mcl-1*^+/−^, *Bcl-2*^+/−^ and *Mcl-1*^+/−^*Bcl-2*^+/−^ males had a significant decrease in testicle weights compared to WT males (Fig. 4b), which was most striking in *Mcl-1*^+/−^, as well as *Mcl-1*^+/−^*Bcl-2*^+/−^ mice. To determine whether this difference was due to anomalies in testes development, we performed histological examination on WT, *Mcl-1*^+/−^, *Bcl-2*^+/−^, and *Mcl-1*^+/−^*Bcl-2*^+/−^ age-matched male mice. However, mice of all genotypes examined were fertile and no obvious differences in testes morphology were observed (Fig. 4c).

**Fig. 5:**
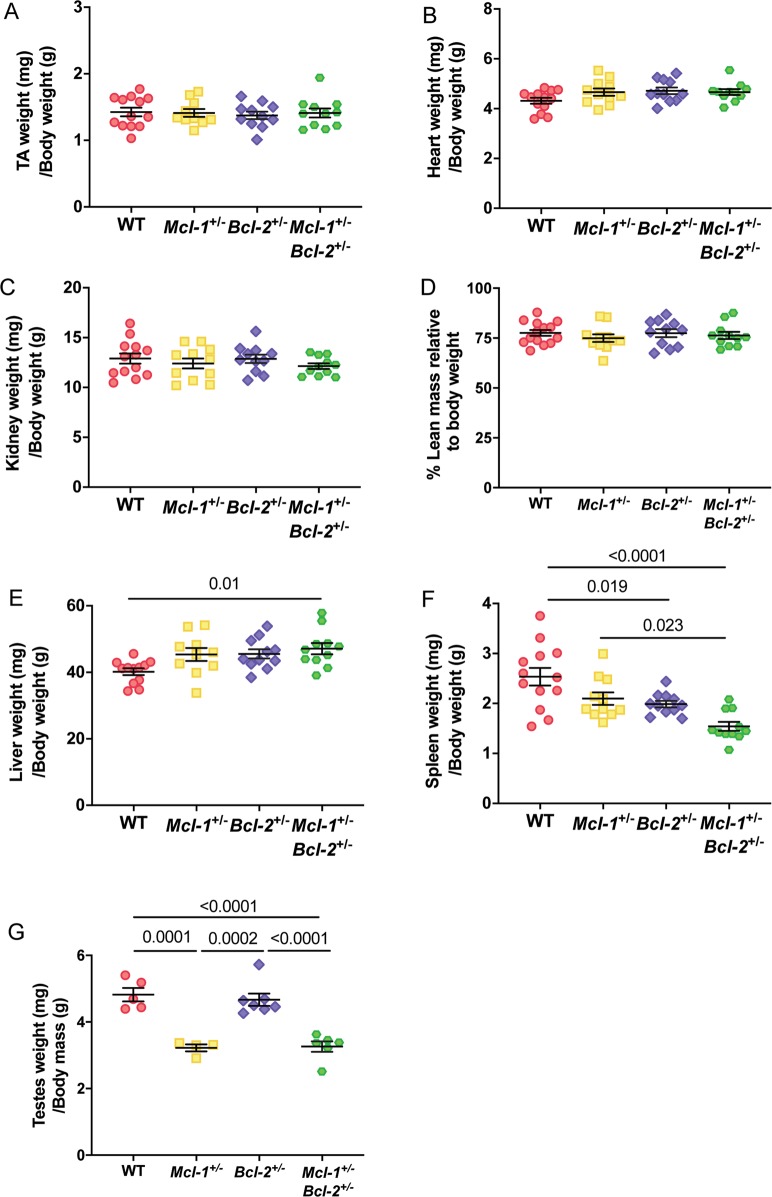
Compound loss of one allele of *Mcl-1* and *Bcl-2* leads to a reduction in tissue weights compared with WT mice. The (**a**) TA muscle, (**b**) heart, (**c**) kidney, (**d**) lean mass, (**e**) liver, (**f**) spleen, and (**g**) testes weights of male mice were expressed in relation to their body weight. Note that testes weight measurements are obtained from a younger age-matched cohort (average age of 280 days) while other organs weights are obtained from older male mice (average age of 509 days). Data represent mean ± S.E.M. and were analysed using one-way ANOVA comparing the mean of each group to every other mean. Statistically significant differences (*P* ≤ 0.05) are indicated in each graph.

Since male mice displayed a more obvious phenotype compared to females, we examined the former in greater detail. When expressed relative to body weight, the TA muscle, heart, kidney weights and total lean mass (all of which were reduced in *Mcl-1*^+/−^*Bcl-2*^+/−^ males) were no longer significantly different between any of the genotypes (Fig. 5a–d). Interestingly, liver weights, which were unaffected by genotype (Fig. 3i), were increased in *Mcl-1*^+/−^*Bcl-2*^+/−^ males after normalisation to body weight (Fig. 5e). Spleen (Fig. 5f) and testes weights (Fig. 5g) remained significantly lower even after normalisation to body weight.

### Histological analysis of major organs revealed no significant differences in the spleen and liver of mice with different genotypes

To further examine the spleen and liver, histological analysis was performed on tissue samples from age-matched mice of the different genotypes (Supplementary Fig. 1). Across all genotypes, we observed varying levels of fat in the liver along with perivascular infiltration, as well as an expansion of the lymphoid areas in the spleen in a proportion of the animals. However, no obvious differences were found between groups. Since the age of the cohort examined was over a year old, these observations were most likely attributable to aging.

### WT, *Mcl-1*^+/−^, *Bcl-2*^+/−^, and *Mcl-1*^+/−^*Bcl-2*^+/−^ mice display similar levels of serum testosterone and IGF-1 levels

**Fig. 6:**
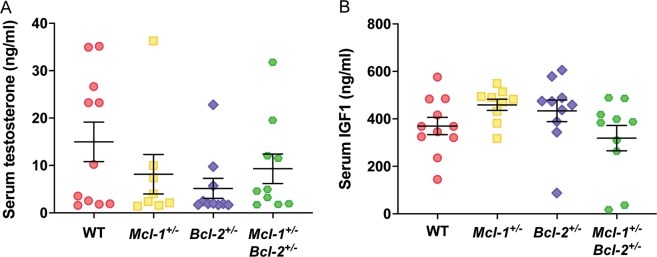
Testosterone and IGF-1 levels are similar between mice of the different genotypes. **a** Serum testosterone and (**b**) IGF1 levels in male mice. Data represent mean ± S.E.M. and were analysed using one-way ANOVA comparing the mean of each group to every other mean. No significant differences were observed between the groups.

The testes produce anabolic hormones, such as testosterone, that have a major role in muscle and bone growth. We considered that the markedly reduced testes weight, and potentially a concomitant reduction in testosterone production, may be a primary driver of the abnormally reduced growth in male *Mcl-1*^+/−^*Bcl-2*^+/−^ mice. However, we observed no differences in serum testosterone levels between mice of any of the genotypes tested (Fig. 6a). Similarly, no differences in IGF1, another major anabolic hormone, were observed between mice of the different genotypes (Fig. 6b).

## Discussion

We have shown that *Mcl-1*^+/−^*Bcl-2*^+/−^ mice, and to a lesser extent *Mcl-1*^+/−^ and *Bcl-2*^+/−^ mice, are smaller than their WT counterparts. While this effect was apparent in females, it was markedly more pronounced in males. When we first noted that *Mcl-1*^+/−^*Bcl-2*^+/−^ animals appeared smaller than single heterozygote and WT animals, we hypothesised that this may have been due to a reduction in fat mass. However, EchoMRI analysis revealed that fat mass was similar between WT, *Mcl-1*^+/−^, *Bcl-2*^+/−^, and *Mcl-1*^+/−^*Bcl-2*^+/−^ mice, and the reduction in body weight was entirely attributable to lower lean mass. Consistently, we also observed reductions in the weights of numerous tissues in *Mcl-1*^+/−^*Bcl-2*^+/−^ mice. Importantly, *Mcl-1*^+/−^*Bcl-2*^+/−^ males had a lower naso-anal length and tibia length compared to WT controls. When expressed relative to body weight, differences in lean mass, as well as weights of the heart, kidney and TA muscle were not significant between the genotypes examined. Collectively, our data indicate that *Mcl-1*^+/−^*Bcl-2*^+/−^ mice, and to a lesser extent *Mcl-1*^+/−^ and *Bcl-2*^+/−^ mice have a reduced organismal size compared with their WT counterparts and this appears to be due to a reduction in cell numbers per tissue (rather than a decrease in cell size). Interestingly, the spleen and testes of *Mcl-1*^+/^*Bcl-2*^+/−^ mice remained significantly smaller compared to control animals even when expressed relative to overall body weight.

The anti-apoptotic roles of MCL-1 and BCL-2 in immune cell survival have been well documented, and it is therefore not unexpected that the spleen weights were reduced in *Mcl-1*^+/−^ and *Bcl-2*^+/−^ mice^9,12^, and further reduced in *Mcl-1*^+/−^*Bcl-2*^+/−^ double heterozygotes (Fig. 3g). Interestingly, the effects of *Mcl-1* and/or *Bcl-2* deficiency on spleen weight was less pronounced in female mice (Fig. 3g). This may suggest that male hormones exacerbate the impairment in cell survival caused by the reduction in BCL-2 and/or MCL-1. Alternatively, female hormones could have a protective effect.

With regards to the reduction in testes weight observed in *Mcl-1*^+/−^*Bcl-2*^+/−^ males, apoptosis is known to play a key role in spermatogenesis, with only 25% of germ cells successfully maturing and the remainder undergoing apoptosis in the early post-natal period^13^. These high rates of apoptosis are essential to establish the appropriate balance between germ cells and Sertoli cells, which support the survival, proliferation, and maturation of the former. Several BCL-2 family members are expressed in the testes and display distinct patterns of expression during testes development^14^. Moreover, previous studies have established important roles for BCL-2 family members in spermatogenesis and testes development. Mice deficient in the pro-apoptotic effector BAX, those lacking BH3-only proteins BIK and BIM, those lacking pro-survival BCL-W, or expressing *a Bcl-2* transgene, all exhibit dysregulated apoptosis, disorganised spermatogenesis, and altered testes weight^14–17^. This suggests that both abnormally increased, as well as abnormally decreased apoptosis can cause defects in spermatogenesis. Our observations of significantly reduced testes weight in *Mcl-1*^+/−^*Bcl-2*^+/−^ males are consistent with these prior findings and suggest an important role for MCL-1 and BCL-2 in testes development.

We have previously shown that the body weight of E19.5 *Mcl-1*^+/−^ pups was significantly lower than WT littermates^6^, and observed that *Mcl-1*^+/−^ mice developed normally and survive into late adulthood^6^. These findings are consistent with the current observations and suggest that the reduction in MCL-1 either alone or in combination with a reduction in BCL-2 likely affects numerous aspects of organismal development and growth. This may be explained by an abnormal increase in apoptosis, leading to reduced cell numbers in early embryogenesis that will carry through to a reduction in overall body cellularity and thus body size. Importantly, with the exception of being smaller in size, *Mcl-1*^+/−^*Bcl-2*^+/−^ double heterozygote mice do not display any overt developmental defects. Collectively, our results demonstrate rate limiting roles for MCL-1 and BCL-2 in organismal growth.

## Methods

### Mice

All experiments were approved by the animal ethics committees of the Walter and Eliza Hall Institute of Medical Research and the Baker Heart and Diabetes Institute and conducted in accordance with the Australian code for the care and use of laboratory animals. *Mcl-1*^+/−^ mice were generated from *Mcl-1*^fl/+^ mice^18^. The *Mcl-1*^+/−^ and *Bcl-2*^+/−^^10^ were all maintained on a C57BL/6 background. All mice were bred and aged at the Walter and Eliza Hall Institute of Medical Research and were maintained in a 14-h light and 10-h dark cycle at 22 °C and fed ad libitum on standard mouse chow. Once the cohorts had aged they were transferred to the Baker Heart and Diabetes Institute, where they were habituated in the new environment for ~2 weeks before assessment of body mass and body composition. Approximately 1 week after the assessment of body composition, mice were culled and body length, tissue weights, and tibia length determined.

### Body composition and mass

Lean and fat mass were determined using a 4-in-1 EchoMRI body composition analyser (EchoMRI^TM^, Houston, TX, USA), as previously described^11^. Standard laboratory scales were used to determine total body mass (Mettler Toledo, Greifensee, Switzerland).

### Statistical analysis

The data presented in Fig. 1 were analysed using a 2-way (genotype x time) analysis of variance (ANOVA) with repeated measures on the time factor. Where a significant interaction effect was observed, statistical analysis of specific pairwise comparisons was conducted using a post-hoc Tukey test. Data in Figs. 2–6 were analysed by 1-way ANOVA using the PRISM software. Where the ANOVA revealed a significant F value, statistical analysis of specific pairwise comparisons was conducted using the Tukey’s test, which adjusts *P* values for multiple comparisons. Where statistical significance was achieved, the adjusted P values are reported within the specific figures.

### Histology

All mouse tissues were fixed in 10% buffered formalin solution and subsequently embedded in paraffin prior to sectioning. Slides were stained with haematoxylin and eosin, then examined and photographed using the CaseViewer Software (3DHISTECH).

### ELISA

ELISAs for testosterone (KGE010) and IGF1 (MG100) were obtained from R&D systems and performed according to the manufacturer’s instructions.

## Supplementary information

Supplementary Figure 1

Supplementary Figure 2
