# Supplementary material for: Combined reduction in the expression of MCL-1 and BCL-2 reduces organismal size in mice
Source: Cell Death Dis. 2020 Mar 13;11(3):185. doi: 10.1038/s41419-020-2376-5 (PMC7070015; doi:10.1038/s41419-020-2376-5)
Supplement: Supplementary file 1 — Supplementary Figure 1 [file 41419_2020_2376_MOESM1_ESM.docx]

**Francine Ke et al**

**Supplementary Text**

**Supplementary Figure 1. Histology of the spleen and liver of WT, *Mcl-1*^+/^, *Bcl-2*^+/-^, and *Mcl-1*^+/^*Bcl-2*^+/-^ male mice.** Sections of the spleen (top panel) and liver (bottom panel) of age-matched male mice from each genotype stained with haematoxylin and eosin. Images of the spleen and liver were obtained at 4.2x magnification and 3x magnification, respectively. Black bars in all histology images represent 500 μm.
