# Supplementary figures and images for: Combined reduction in the expression of MCL-1 and BCL-2 reduces organismal size in mice
Source: Cell Death Dis. 2020 Mar 13;11(3):185. doi: 10.1038/s41419-020-2376-5 (PMC7070015; doi:10.1038/s41419-020-2376-5)

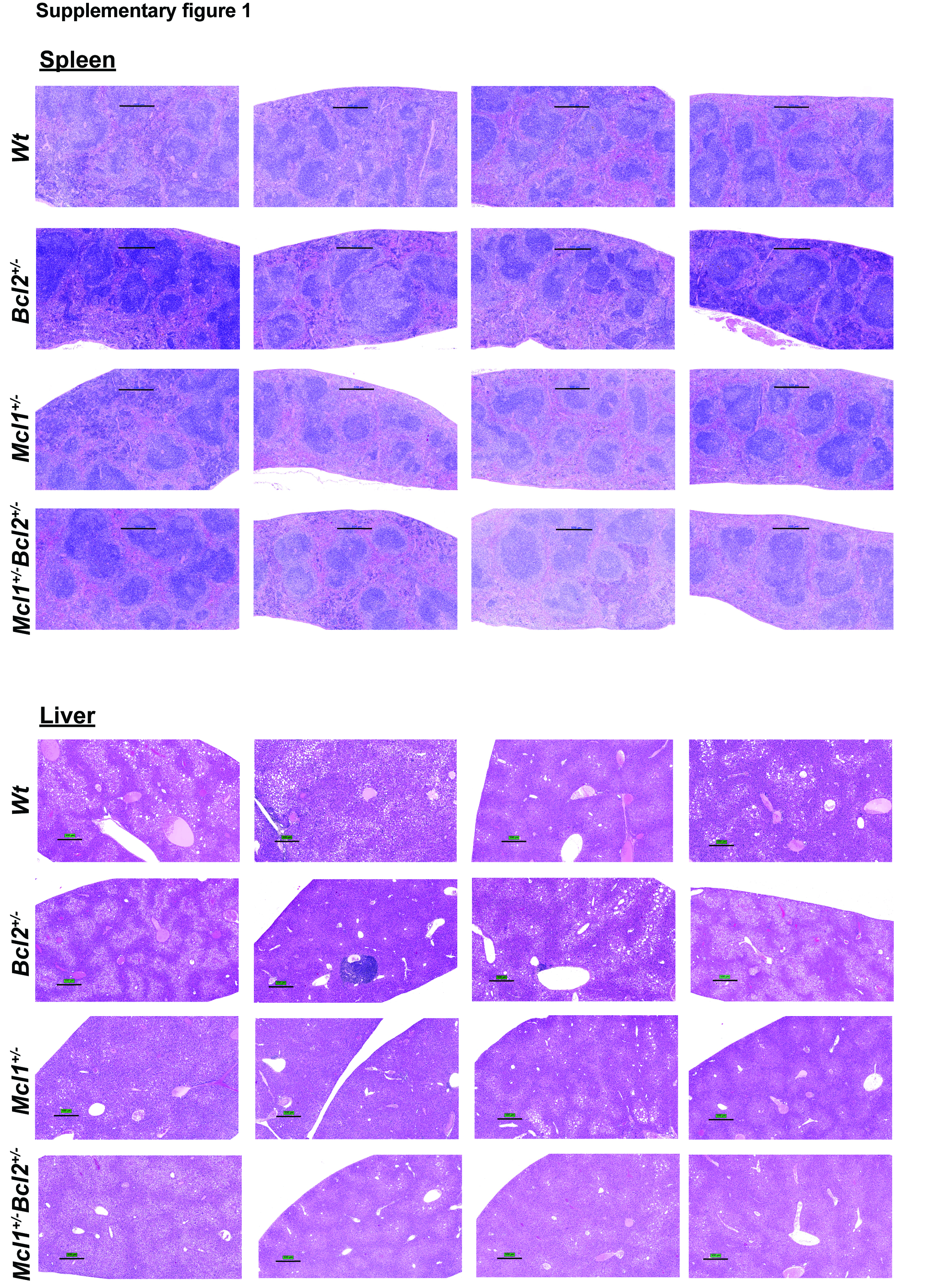

Supplement: Supplementary file 2 — Supplementary Figure 2 [file 41419_2020_2376_MOESM2_ESM.tif]
